# Supplementary material for: Lifestyle and Transcriptional Signatures Associated with Ethnicity/Race-Related Variations in the Functional Connectome
Source: Research (Wash D C). 2026 Mar 24;9:1143. doi: 10.34133/research.1143 (PMC13009535; doi:10.34133/research.1143)
Supplement: Supplementary 1 — Figs. S1 to S15 Tables S1 to S10 [file research.1143.f1.zip › Supplementary_materials_1230_majorRe2.docx]

**SUPPLEMENTARY INFORMATION**

for

Lifestyle and transcriptional signatures associated with
ethnicity/race-related variations in the functional connectome

Han *et al.*

**Contents**

[Supplementary Methods 3](#_Toc218350771)

[Supplementary Figure 1 Ethnicity/race-related variability in the spatial topography of functional brain organization. 4](#_Toc218350772)

[Supplementary Figure 2 Topographic variability of individual-specific parcellations across AA–WA pairs is higher in association cortices than in sensorimotor cortices. 5](#_Toc218350773)

[Supplementary Figure 3 Model accuracy across 40 repeated data splits. 6](#_Toc218350774)

[Supplementary Figure 4 Haufe-transformed weights of the ethnicity/race prediction model. 7](#_Toc218350775)

[Supplementary Figure 5 Comparison of Haufe-transformed weights across different parcellations. 8](#_Toc218350776)

[Supplementary Figure 6 Ethnicity/race-related variability in brain morphometric similarity patterns in the HCP-D dataset. 9](#_Toc218350777)

[Supplementary Figure 7 Association with cortical myelin content. 10](#_Toc218350778)

[Supplementary Figure 8 Network-level Haufe-transformed predictive feature matrices for education, substance use, income, physical activity, social relationships and sleep health measures in the HCP-YA dataset. 11](#_Toc218350779)

[Supplementary Figure 9 Explained variance for the first 15 components across subjects. 12](#_Toc218350780)

[Supplementary Figure 10 Representational similarity analysis (RSA) linking inter-subject dissimilarity in full connectivity matrices and the first principal component. 13](#_Toc218350781)

[Supplementary Figure 11 Predictive model accuracy across 40 data splits based on the first principal component. 14](#_Toc218350782)

[Supplementary Figure 12 Sensitivity analyses of the dimensionality reduction strategy. 15](#_Toc218350783)

[Supplementary Figure 13 Robustness of weighted gene expression maps (PLS1) across multiple intensity-based filtering (IBF) thresholds. 16](#_Toc218350784)

[Supplementary Figure 14 Robustness of weighted gene expression maps (PLS1) across multiple differential stability (DS) thresholds. 17](#_Toc218350785)

[Supplementary Figure 15 Gene expression profiles associated with ethnicity/race-related functional connectivity variability in the HCP-D dataset. 18](#_Toc218350786)

[Supplementary Table 1. Demographic characteristics of participants. 19](#_Toc218350787)

[Supplementary Table 2. Lifestyle characteristics of participants. 20](#_Toc218350788)

[Supplementary Table 3. Representative enriched terms of the PLS1+ genes (Z > 5, DS > 0.1) in the HCP-YA dataset. Log10 (*P_FDR_*) is the FDR adjusted *P*-value in log base 10. *P_spin_* denotes the *P*-value computed using a spin-based ensemble null model that accounts for the effects of gene coexpression and spatial autocorrelation. 21](#_Toc218350789)

[Supplementary Table 4. Representative enriched terms of the PLS1+ genes (*Z* > 5, DS > 0) in the HCP-YA dataset. Log10 (*P_FDR_*) is the FDR adjusted *P*-value in log base 10. *P_spin_* denotes the *P*-value computed using a spin-based ensemble null model that accounts for the effects of gene coexpression and spatial autocorrelation. 22](#_Toc218350790)

[Supplementary Table 5. Representative enriched terms of the PLS1+ genes (Z > 5, DS > 0.2) in the HCP-YA dataset. Log10 (*P_FDR_*) is the FDR adjusted *P*-value in log base 10. *P_spin_* denotes the *P*-value computed using a spin-based ensemble null model that accounts for the effects of gene coexpression and spatial autocorrelation. 23](#_Toc218350791)

[Supplementary Table 6. Representative enriched terms of the PLS1- genes (*Z* < -5, DS > 0.1) in the HCP-YA dataset. Log10 (*P_FDR_*) is the FDR adjusted *P*-value in log base 10. *P_spin_* denotes the *P*-value computed using a spin-based ensemble null model that accounts for the effects of gene coexpression and spatial autocorrelation. 24](#_Toc218350792)

[Supplementary Table 7. Representative enriched terms of the PLS1- genes (*Z* < -5, DS > 0) in the HCP-YA dataset. Log10 (*P_FDR_*) is the FDR adjusted *P*-value in log base 10. *P_spin_* denotes the *P*-value computed using a spin-based ensemble null model that accounts for the effects of gene coexpression and spatial autocorrelation. 25](#_Toc218350793)

[Supplementary Table 8. Representative enriched terms of the PLS1- genes (*Z* < -5, DS > 0.2) in the HCP-YA dataset. Log10 (*P_FDR_*) is the FDR adjusted *P*-value in log base 10. *P_spin_* denotes the *P*-value computed using a spin-based ensemble null model that accounts for the effects of gene coexpression and spatial autocorrelation. 26](#_Toc218350794)

[Supplementary Table 9. Representative enriched terms of the PLS1+ genes (*Z* > 5, DS > 0.1) in the HCP-D dataset. Log10 (*P_FDR_*) is the FDR adjusted *P*-value in log base 10. *P_spin_* denotes the *P*-value computed using a spin-based ensemble null model that accounts for the effects of gene coexpression and spatial autocorrelation. 27](#_Toc218350795)

[Supplementary Table 10. Representative enriched terms of the PLS1- genes (*Z* < -5, DS > 0.1) in the HCP-D dataset. Log10 (*P_FDR_*) is the FDR adjusted *P*-value in log base 10. *P_spin_* denotes the *P*-value computed using a spin-based ensemble null model that accounts for the effects of gene coexpression and spatial autocorrelation. 28](#_Toc218350796)

[Equation for structural equation models 29](#_Toc218350797)

Supplementary Methods

**Quality control**

This study utilized two publicly available datasets from the HCP (Human Connectome Project): the HCP-Young Adult (HCP-YA, S1200 release)[1] and HCP-Development (HCP-D, lifespan release 2.0)[2]. The initial sample of HCP-YA consisted of 1,094 healthy young adults (age 22–37 years), including twins and siblings. The initial sample of HCP-D comprised 652 healthy participants (age 5–21 years), also encompassing diverse family structures.

To ensure data quality, we implemented a multi-step quality control pipeline for the functional MRI data:
(***i***) **Motion censoring**
Volumes exhibiting excessive head motion were identified using a root-mean-square framewise displacement (RMS) threshold of > 0.2 mm or a root mean square of voxel-wise differentiated signal (DVARS) threshold of > 75.
(***ii***) **Temporal masking**
To account for the temporal spread of motion artifacts, one frame preceding and two frames following any suprathreshold volume were flagged as censored. Additionally, any uncensored data segments lasting fewer than five contiguous volumes were discarded.
(*iii*) **Run exclusion**
The blood-oxygen-level-dependent (BOLD) runs were entirely removed if more than 50% of the frames were flagged as censored.

Of the 1,029 subjects who survived the motion censoring in the HCP-YA dataset, an additional 81 participants were excluded due to missing data for the behavioral measures or confounding variables, resulting in 948 subjects. The 948 participants included 721 white Americans (WA; 62 Hispanic + 659 non-Hispanic), 129 African Americans (AA), 59 Asian/native Hawaiian/other Pacific Islander participants, 2 American Indians/Alaskan natives, 22 participants with mixed ethnicities/races, and 15 participants with unknown or nonreported ethnic/racial information. 28 AA participants were further excluded from subsequent analyses because their demographic and motion profiles (age, sex, RMS) were markedly different from those of the available WA participants (assessed via Hungarian matching)[3]. The final sample consisted of 721 WA and 101 AA participants, matching the HCP-YA subset utilized in a previous study[3].

Following the same rigorous quality control procedures described above, 404 WA and 68 AA participants in the HCP-D dataset passed motion censoring and were retained as the validation dataset for all neuroimaging analyses. It is worth noting that comprehensive lifestyle behavior data were available only for a subset of HCP-D participants (primarily those younger than 17 years). To avoid substantially reducing the AA sample size, lifestyle-related behavioral analyses were not performed on the HCP-D cohort.

The final list of included subjects for both cohorts has been made publicly available on our GitHub repository (<https://github.com/TianyiYanLab/Ethnicity_Race_Diversity>).

Supplementary Figure 1

**
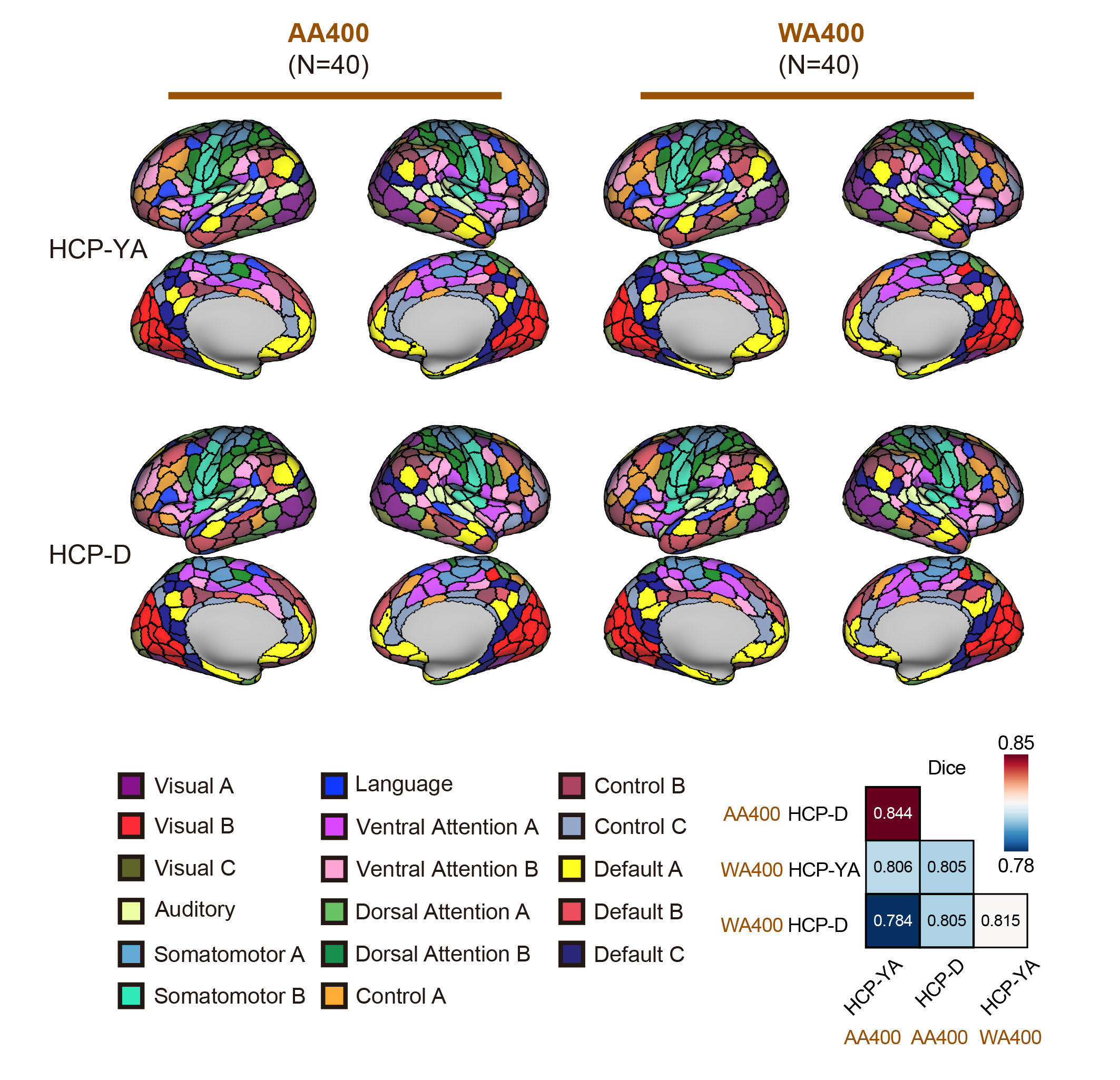
**

**Fig. S1 Ethnicity/race-related variability in the spatial topography of functional brain organization.** Group-level areal parcellation maps (AA400 and WA400) were generated from 40 independent AA and WA individuals’ individualized parcellations, respectively, by assigning each cortical vertex to its most likely parcel. The group-level AA400 and WA400 parcellations capture both shared and unique topographical features. Notably, parcellation maps within the same ethnic/racial group across datasets showed high similarity (Dice = 0.844 for AA; 0.815 for WA), whereas the similarity between AA and WA maps across datasets was substantially lower (Dice = 0.784).

Supplementary Figure 2

**
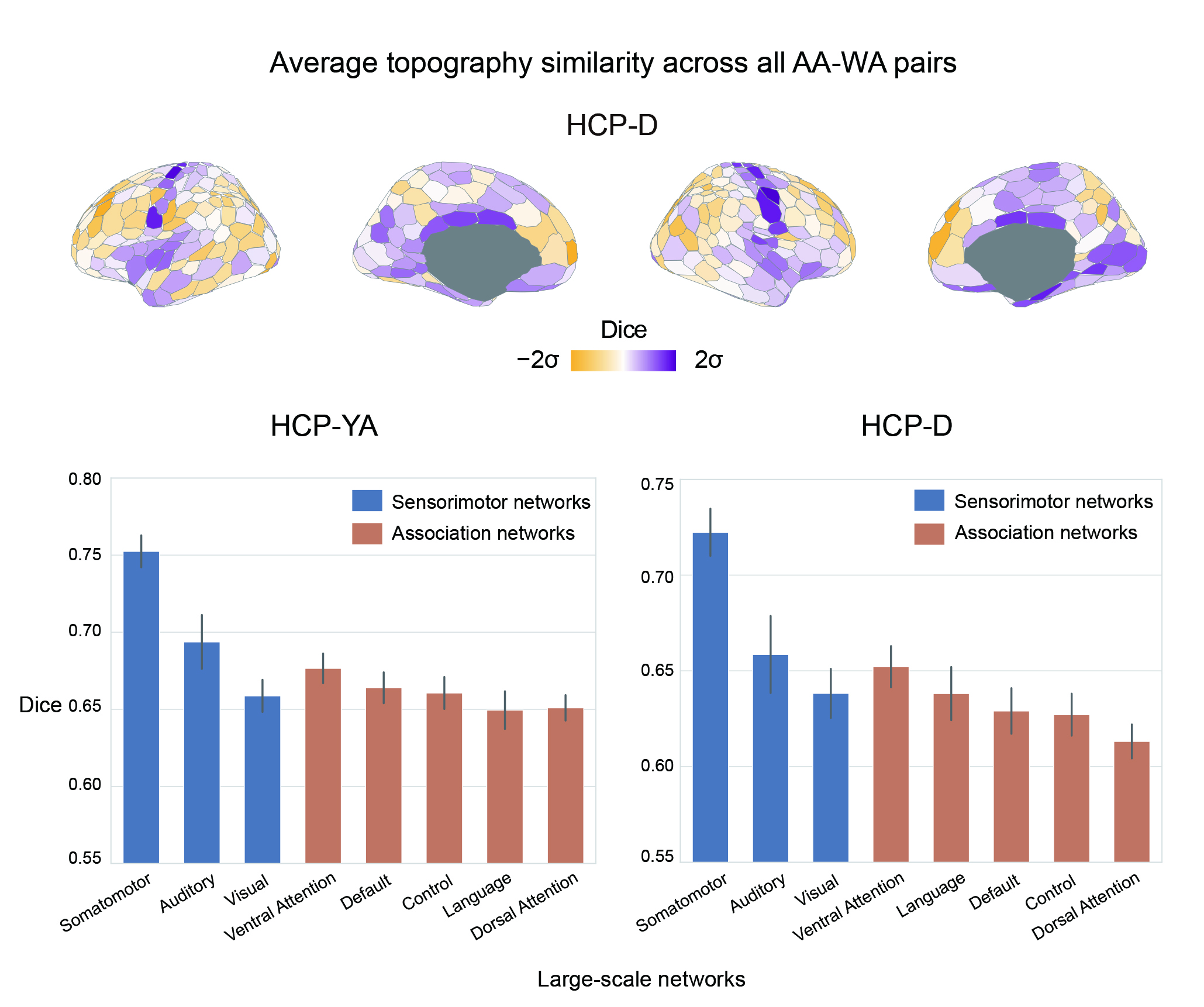
**

**Fig. S2** **Topographic variability of individual-specific parcellations across AA–WA pairs is higher in association cortices than in sensorimotor cortices.** Ethnicity/race-related topographic variability was more pronounced in association cortices than in unimodal sensorimotor cortices. The Yeo atlas[4] is divided into association cortical networks, including default, control, dorsal attention, ventral attention and language networks, and sensorimotor cortical networks, including auditory, somatomotor and visual networks. Each bar shows the average Dice value across regions within a network, with error bars representing the standard error of the mean.

Supplementary Figure 3


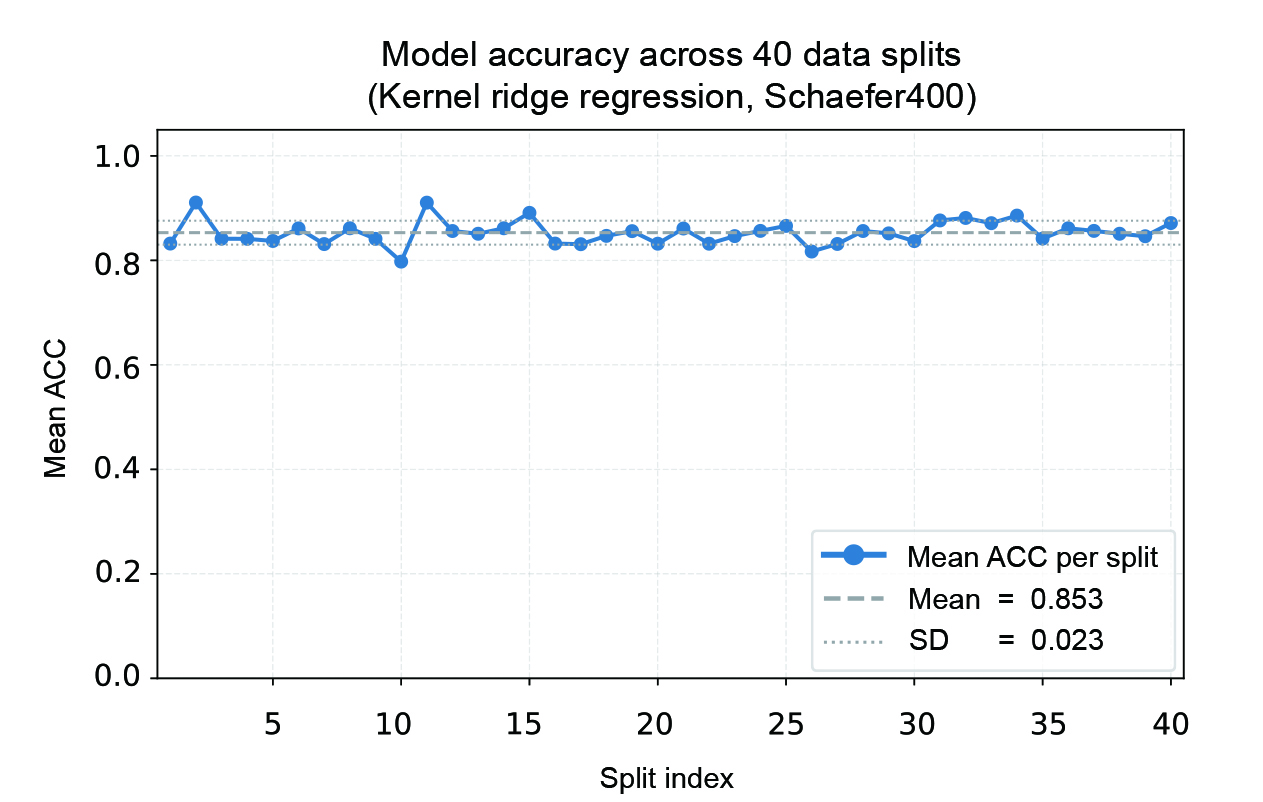


**Figure. S3. Model accuracy across 40 repeated data splits.** Mean prediction accuracy obtained from 40 repetitions of the cross-validation procedure using kernel ridge regression (KRR). Each point represents the prediction accuracy of one data split. The distribution of accuracies is highly stable (mean = 0.853; standard deviation = 0.023).

Supplementary Figure 4

**
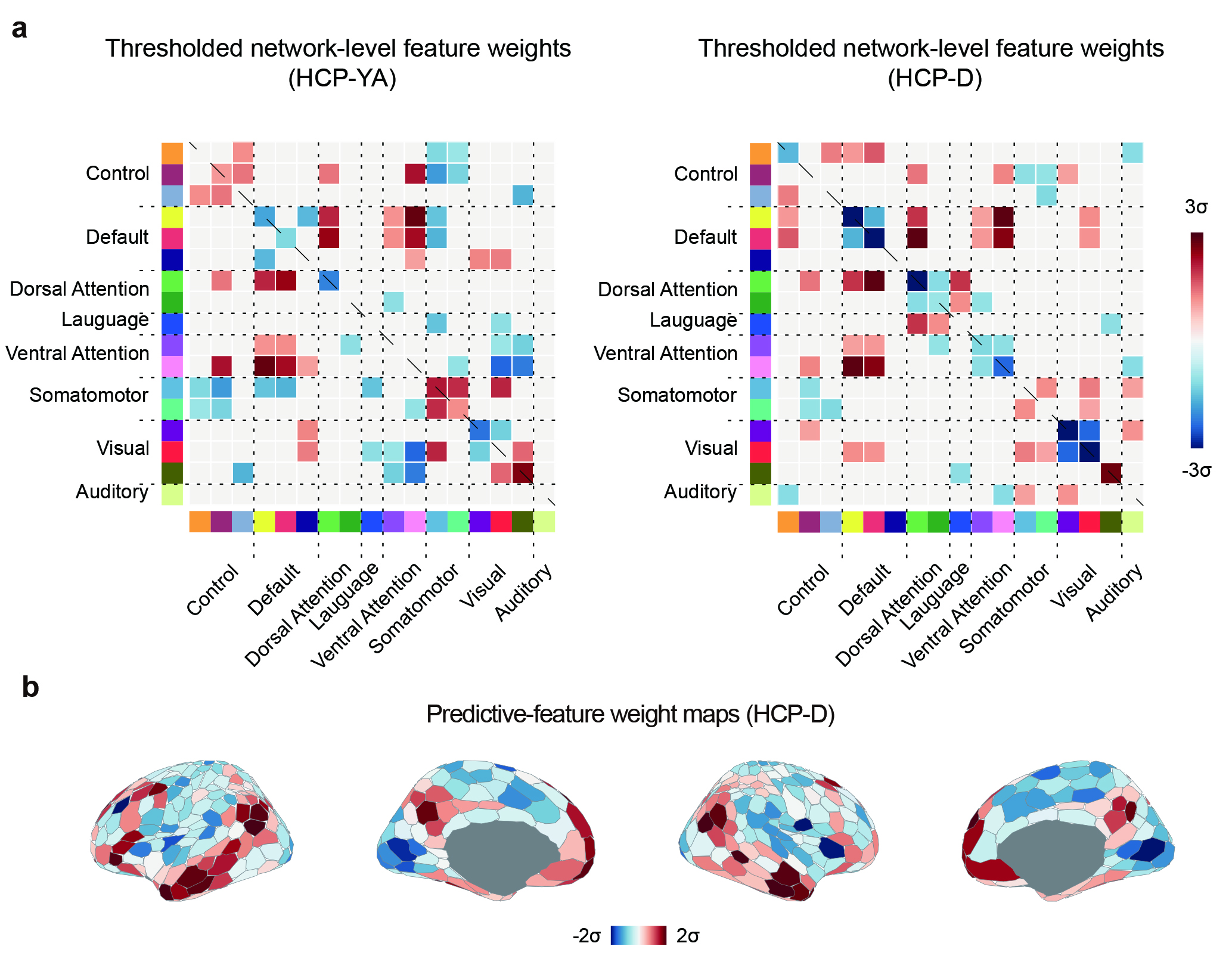
**

**Fig. S4 Haufe-transformed weights of the ethnicity/race prediction model.** a, Thresholded Haufe-transformed predictive feature matrices for both datasets. The top quartile of the absolute weight values from the original matrices was retained, and the corresponding original values was displayed. b, The Haufe-transformed weights of each region were averaged to generate ethnicity/race-predictive weight maps in the HCP-D dataset.

Supplementary Figure 5

**
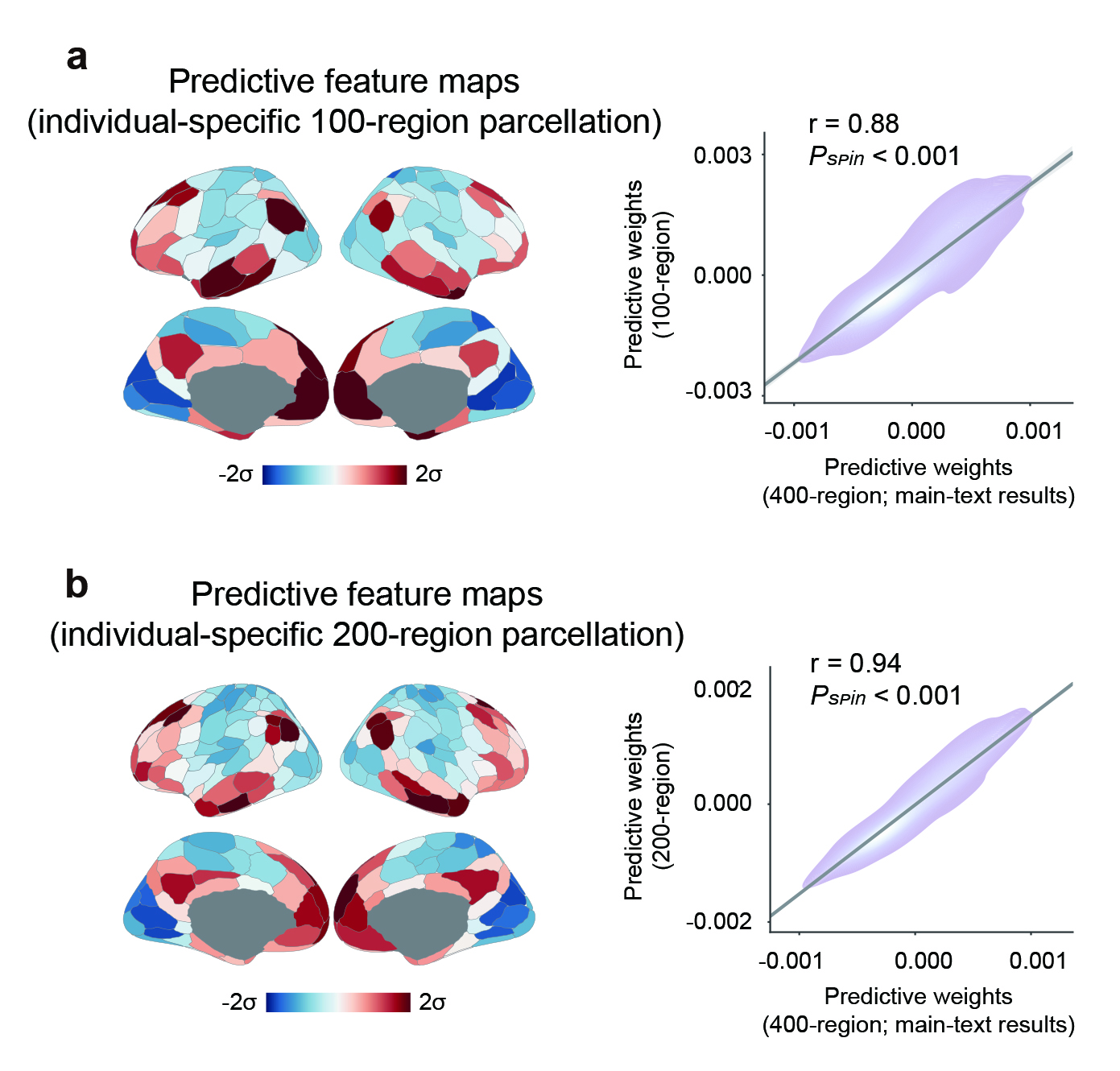
**

**Fig. S5. Comparison of Haufe-transformed weights** **across different parcellations. a,** Correspondence between predictive feature maps obtained using the individual-specific 100-region and individual-specific 400-region parcellations (*r* = 0.88, *P_spin_* < 0.001, two-sided). **b,** Correspondence between predictive feature maps obtained using the individual-specific 200-region and individual-specific 400-region parcellations (*r* = 0.94, *P_spin_* < 0.001, two-sided). All individual-specific parcellations were generated using the gradient-infused multisession hierarchical Bayesian model (gMS-HBM).

Supplementary Figure 6


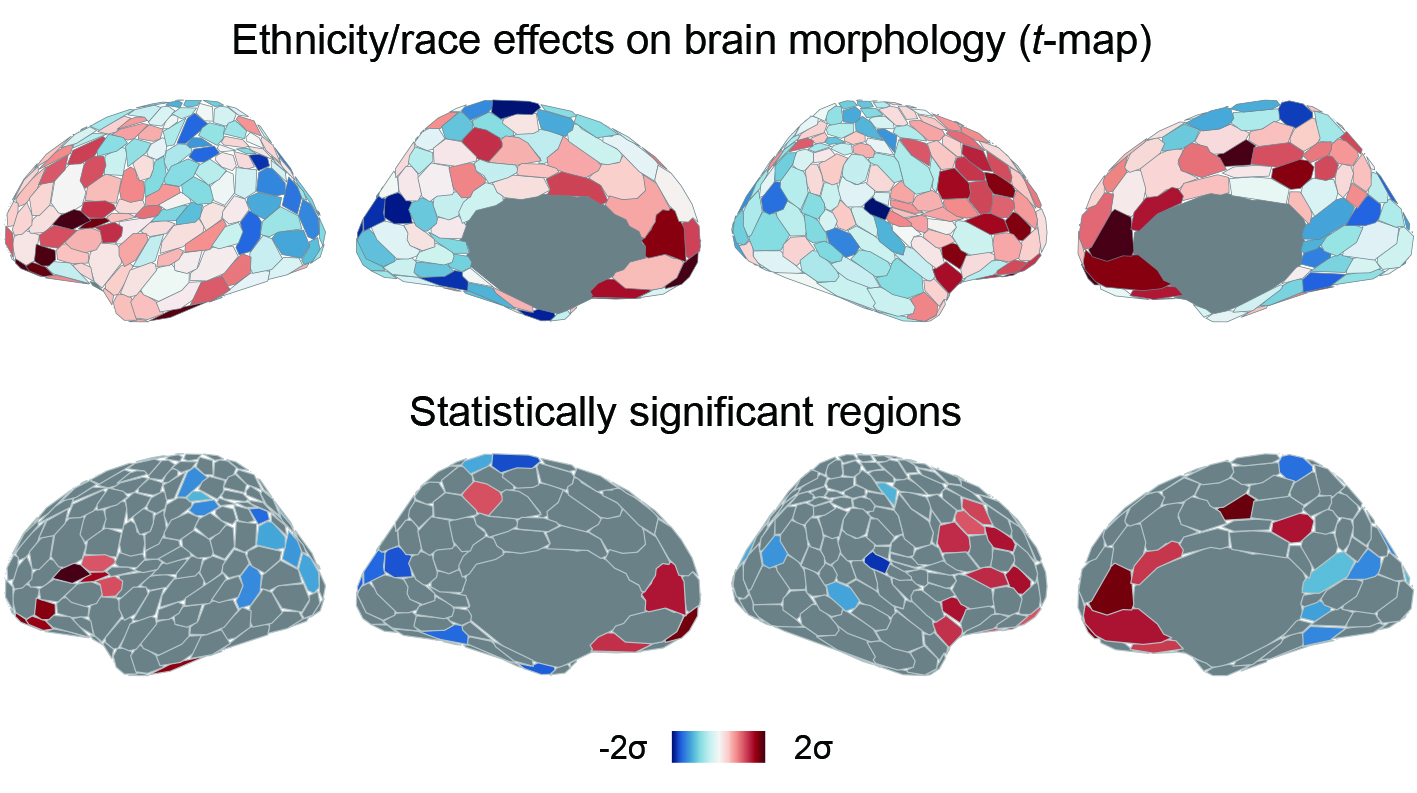


**Fig. S6** **Ethnicity/race-related variability in brain morphometric similarity patterns in the HCP-D dataset.** Top panel: Comparison (*t*-map) of regional morphometric similarity patterns across ethnic/racial groups. Bottom panel: False discovery rate (FDR) correction with *P* < 0.05 for multiple comparisons across 400 regions revealed statistically significant ethnicity/race-related effects in cortical morphometry.

Supplementary Figure 7

**
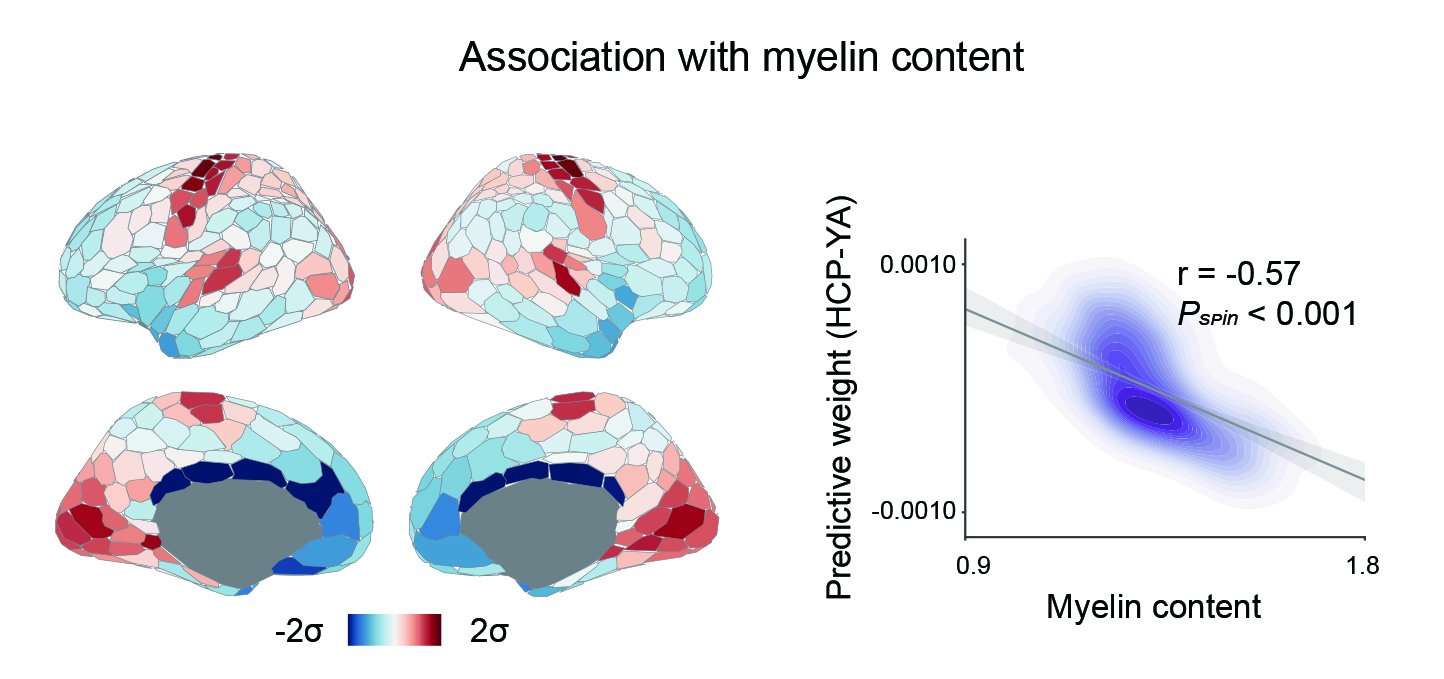
**

**Fig. S7** **Association with cortical myelin content.** Ethnicity/race-predictive weights were negatively correlated with the distribution of cortical myelin content (*r* = -0.57, *P*_spin_ < 0.001, two-sided), which was estimated from the T1w/T2w ratio.

Supplementary Figure 8


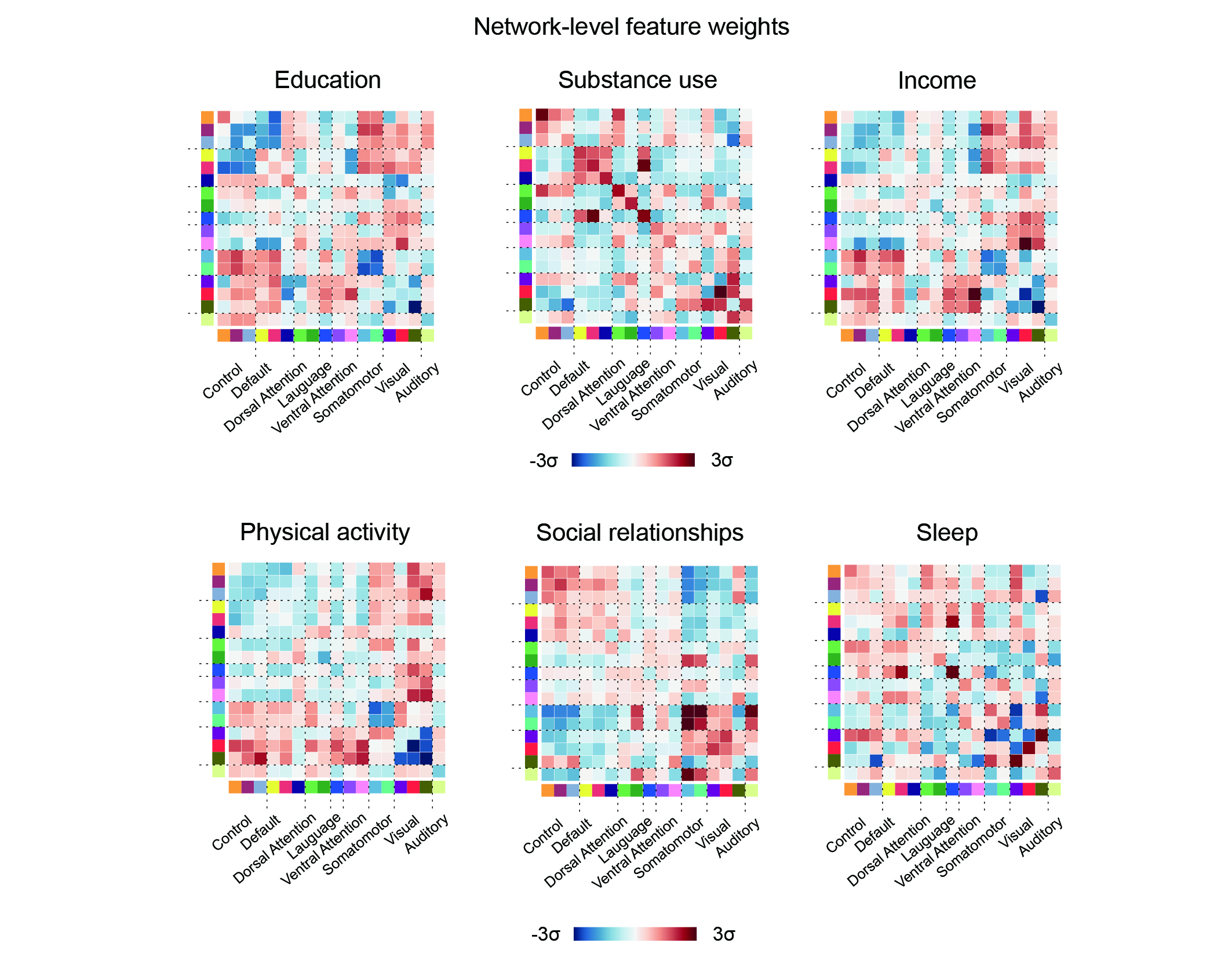


**Fig. S8 Network-level Haufe-transformed predictive feature matrices for education, substance use, income, physical activity, social relationships and sleep health measures in the HCP-YA dataset.** Regional pairwise feature weights were averaged to the network level based on the Yeo networks. For visualization, actual values were subjected to *Z* score standardization.

Supplementary Figure 9


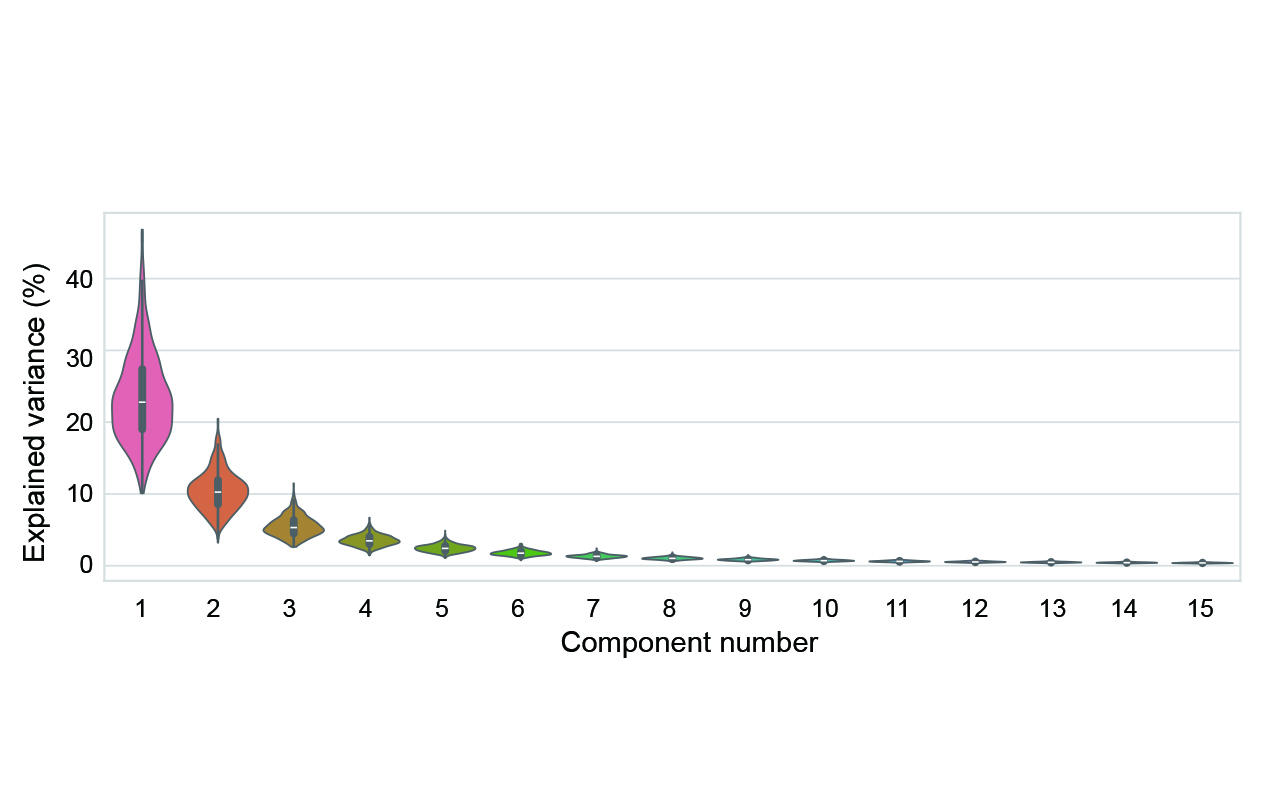


**Fig. S9. Explained variance for the first 15 components across subjects.** Each violin plot displays the distribution of individual-level (N = 822) explained variance for the corresponding component; the internal box indicates the median and interquartile range. Data were computed from per-subject principal component analysis (PCA) applied to the full functional connectivity matrices.

Supplementary Figure 10


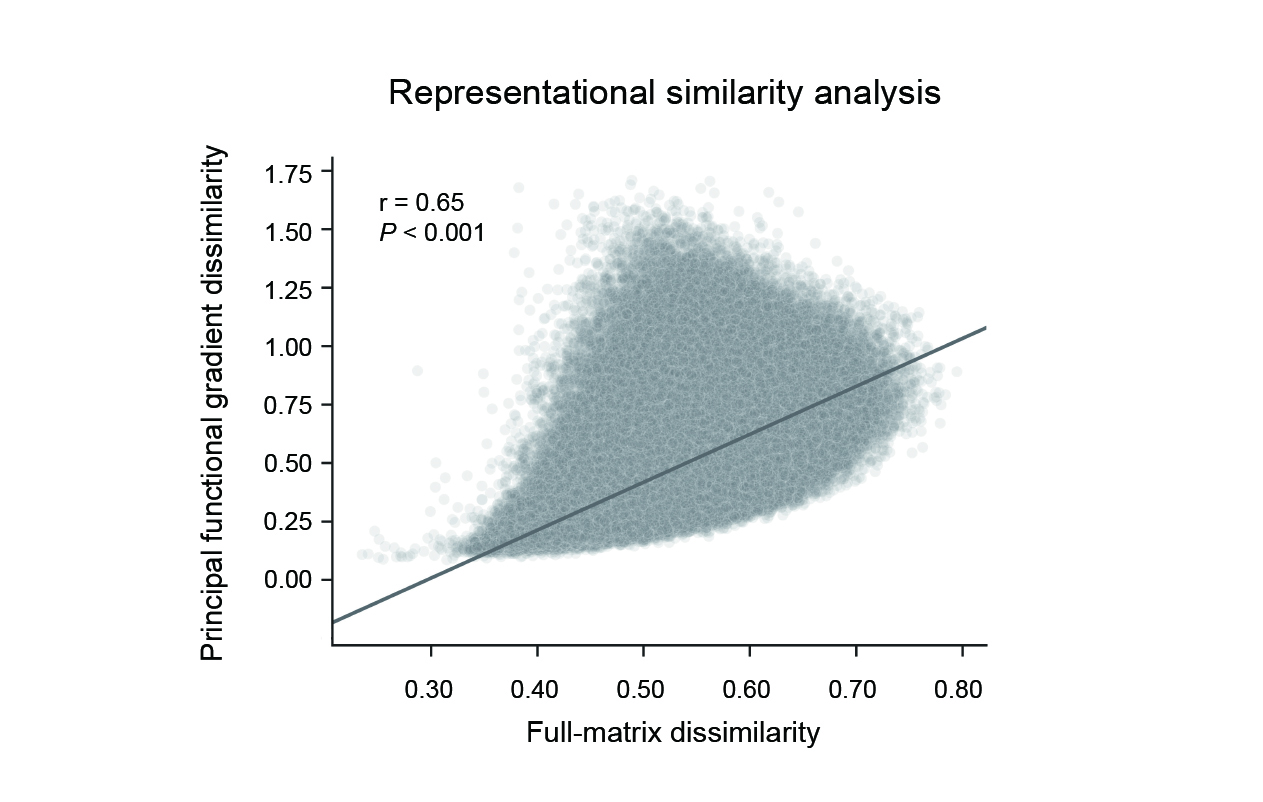


**Fig. S10. Representational similarity analysis (RSA) linking inter-subject dissimilarity in full connectivity matrices and the first principal component.** For each pair of subjects, the Spearman distance (1 – Spearman’s r) was computed from the upper-triangular edges of the full matrix and from the first principal component derived from PCA. Each point represents one subject pair (N = 337,431).

Supplementary Figure 11


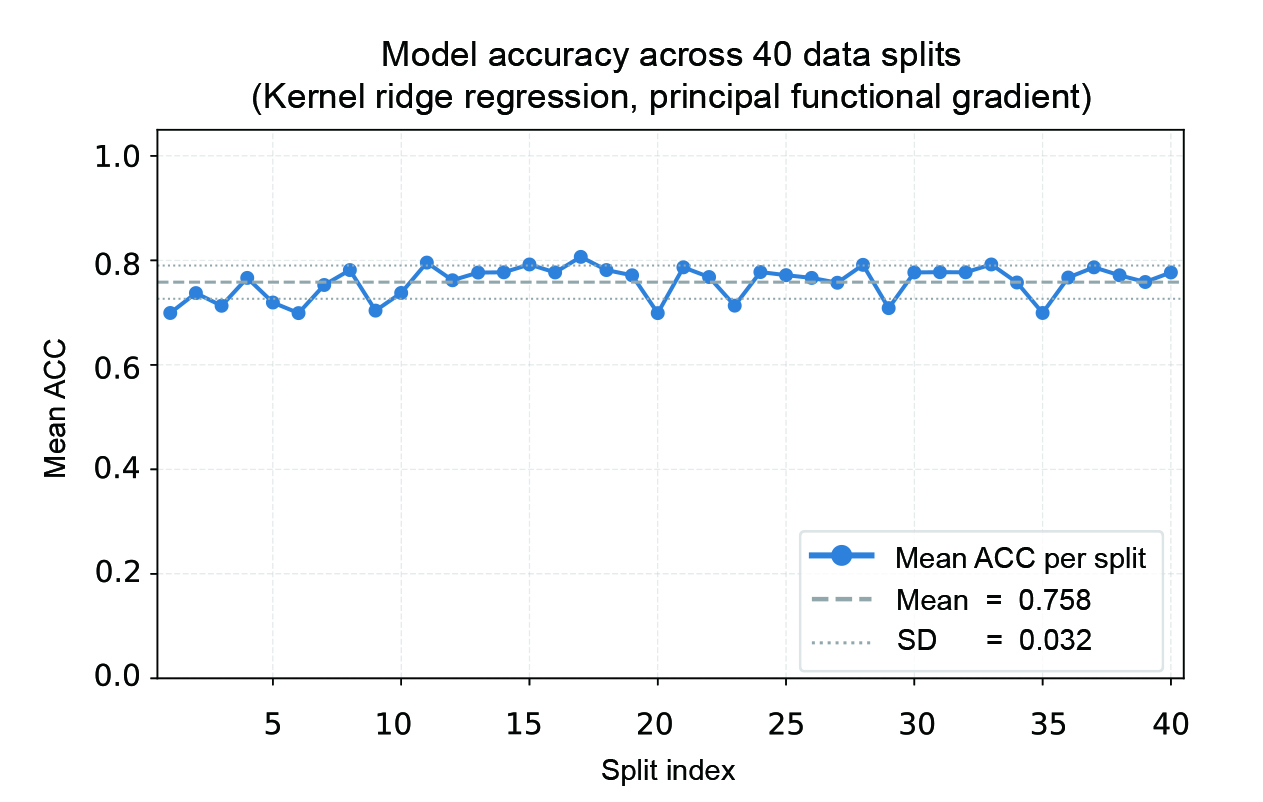


**Fig. S11. Predictive model accuracy across 40 data splits based on the first principal component.** Kernel ridge regression (KRR) models were trained using the first principal component derived from PCA to predict individuals’ self-reported ethnicity/race. The prediction analysis followed the identical framework as in the main text. The distribution of prediction accuracies across 40 repetitions demonstrates stable performance (mean = 0.758, standard deviation = 0.032). Importantly, using the first principal component alone resulted in an approximate 11.1% relative reduction in prediction accuracy compared with models based on the full data, indicating substantial retention of ethnicity/race-related information.

Supplementary Figure 12


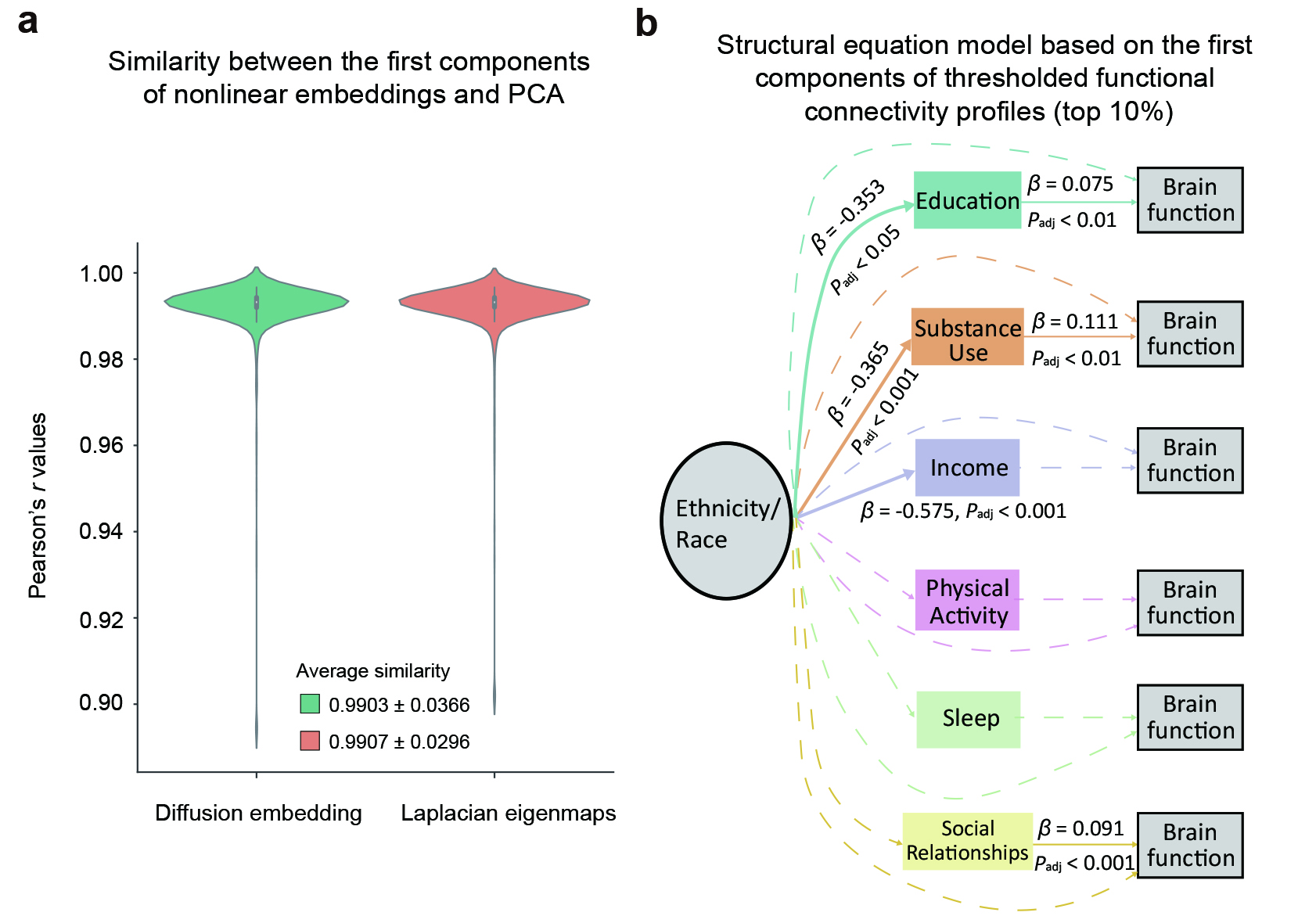


**Fig. S12. Sensitivity analyses of the dimensionality reduction strategy.** **a,** Violin plots show the distribution of correlations between the first component derived from PCA and those from two nonlinear embedding methods (diffusion embedding and Laplacian eigenmaps) across subjects. Both nonlinear approaches yielded virtually identical first components to PCA (*r* > 0.99). **b,** A structural equation model was fitted for each lifestyle factor, thereby revealing the relationships between ethnicity/race, lifestyle and brain functional organization (first principal component of the top-10% thresholded functional connectivity profiles), while controlling for age, sex, RMS and ICV. Edges represent regression coefficients (*β* values). Significant links (FDR-corrected *P* < 0.05, two-sided) are shown as solid lines; non-significant links as dashed lines. Results demonstrated that the mediating roles of education and substance use remained significant (95% bootstrap CI did not include 0).

Supplementary Figure 13

**
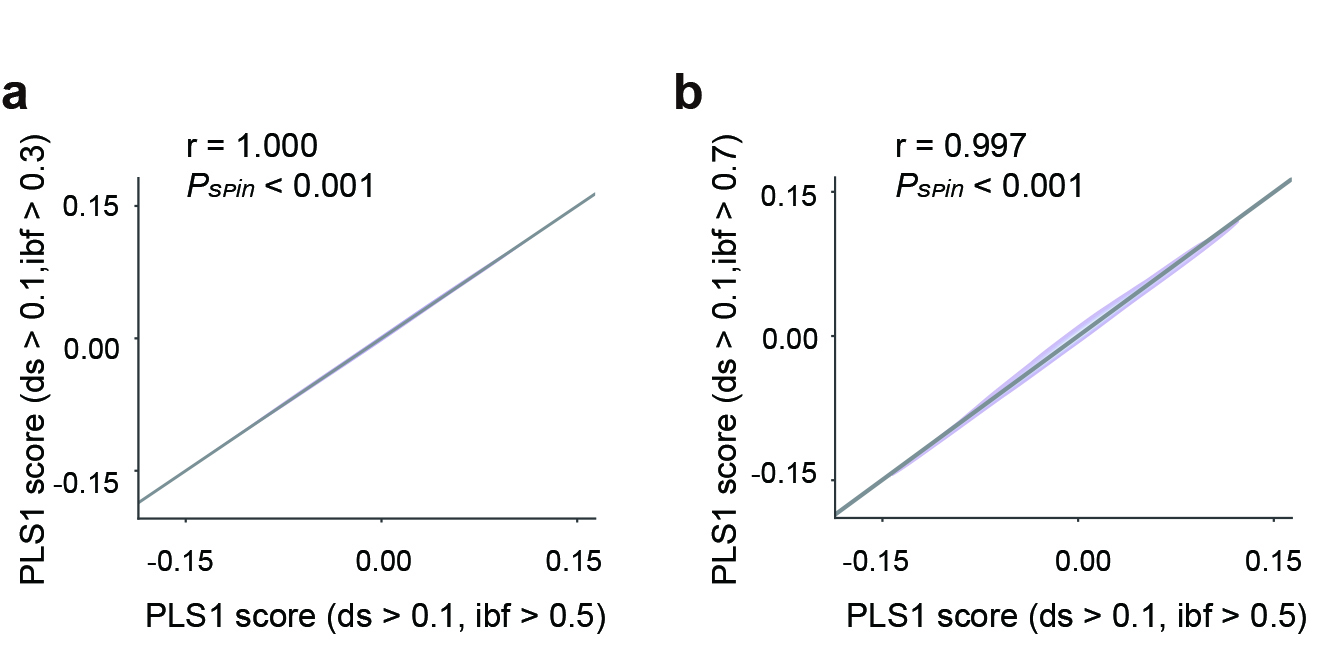
**

**Fig. S13 Robustness of weighted gene expression maps (PLS1) across multiple intensity-based filtering (IBF) thresholds. a,** Correspondence between the PLS1 weighted gene expression map computed using genes with IBF > 0.3 and the map based on the IBF > 0.5 used in the main analysis (*r* = 1.000, *P_spin_* < 0.001, two-sided). **b,** Correspondence between the PLS1 weighted gene expression map computed using genes with IBF > 0.7 and the PLS1 map based on the IBF > 0.5 genes (*r* = 0.997, *P_spin_* < 0.001, two-sided)**.**

Supplementary Figure 14

**
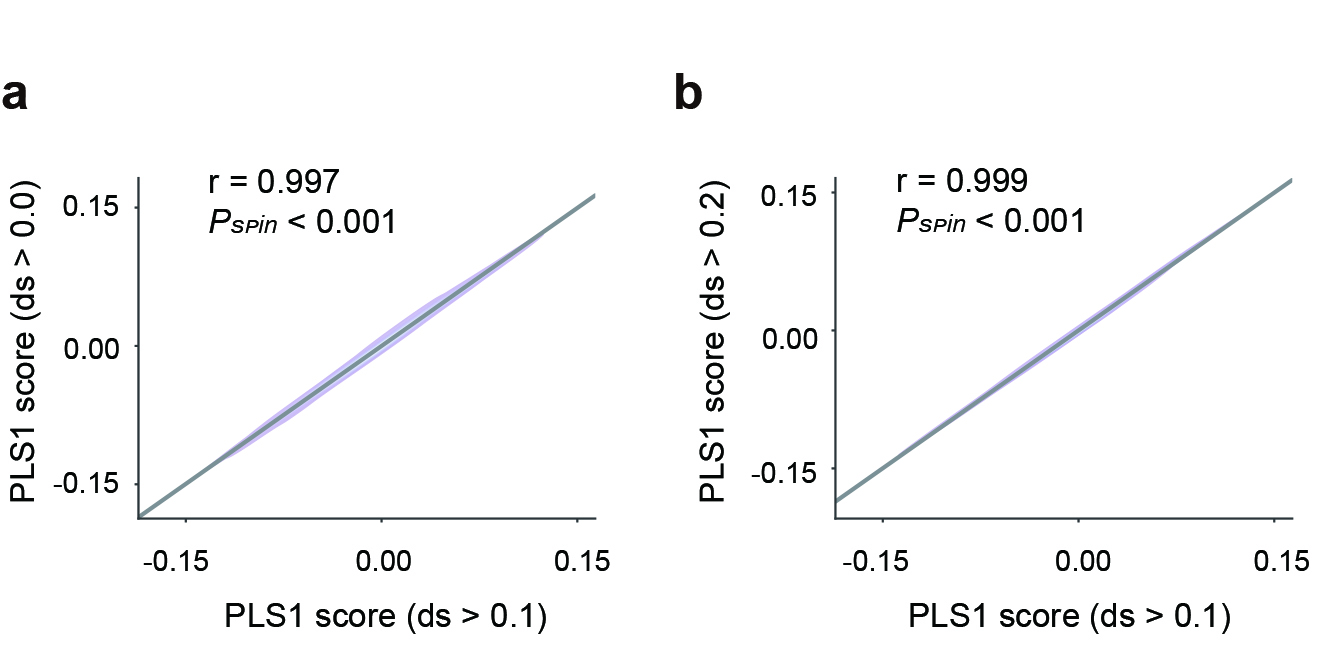
**

**Fig. S14 Robustness of weighted gene expression maps (PLS1) across multiple differential stability (DS) thresholds. a,** Correspondence between the PLS1 weighted gene expression map computed using genes with DS > 0 and the map based on the DS > 0.1 used in the main analysis (*r* = 0.997, *P_spin_* < 0.001, two-sided). **b,** Correspondence between the PLS1 weighted gene expression map computed using genes with DS > 0.2 and the PLS1 map based on the DS > 0.1 genes (*r* = 0.999, *P_spin_* < 0.001, two-sided).

Supplementary Figure 15


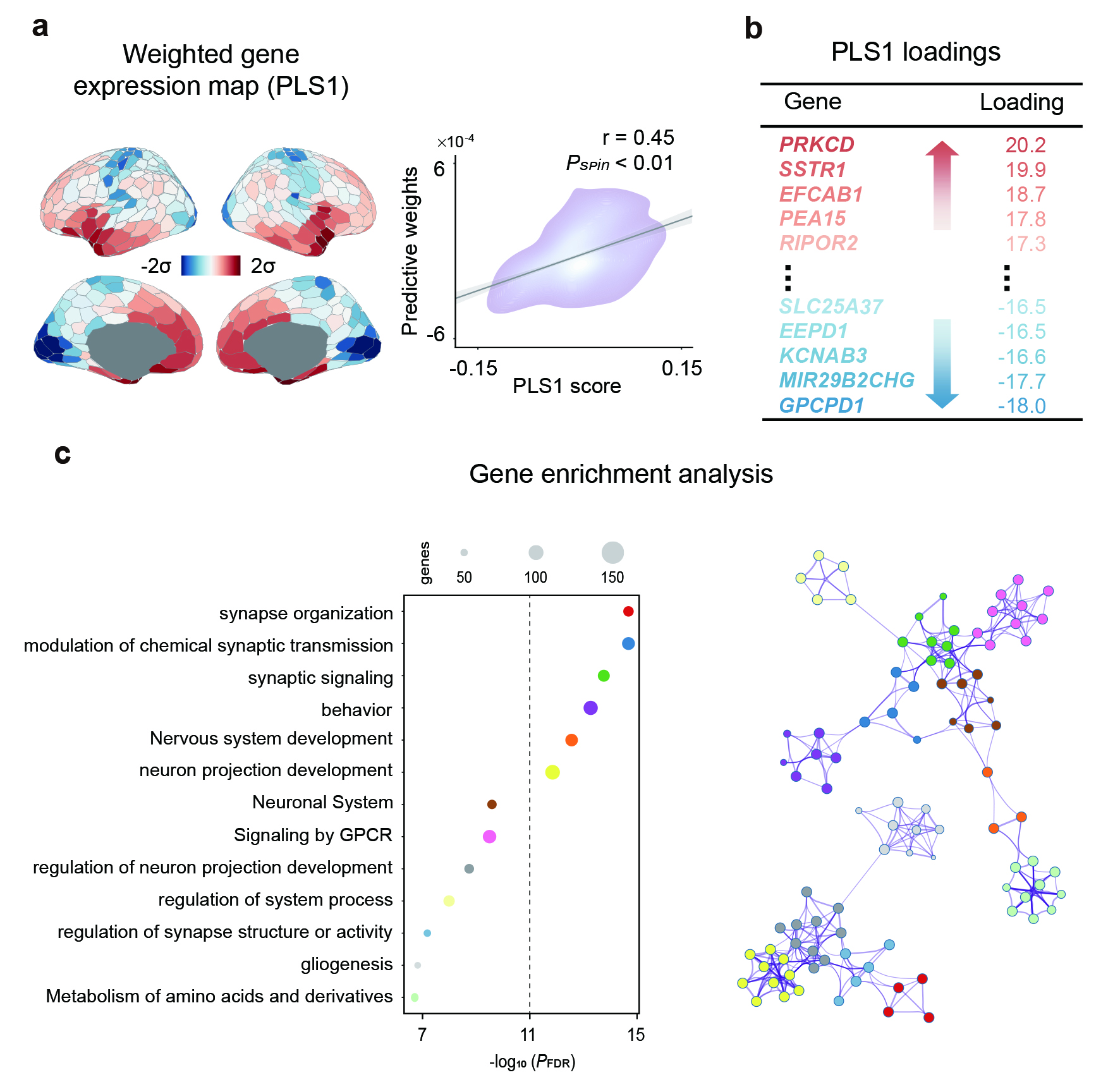


**Fig. S15 Gene expression profiles associated with ethnicity/race-related functional connectivity variability in the HCP-D dataset. a,** Weighted cortical gene expression map of the regional PLS1 scores (top panel). Scatterplot showing the relationship between the PLS map and the Haufe-transformed predictive weight map (bottom panel, *r* = 0.45, *P_spin_* < 0.01, two-sided). **b,** Genes were ranked according to their loadings on PLS1. **c,** Left panel: Representative enriched terms of PLS1+ genes (*Z* > 5). Circle size represents the number of genes within a given ontology term, and the multi-test FDR-adjusted *P* values are plotted as log10-transformed values. Right panel: Metascape network plot of enriched terms, capturing intra-cluster and inter-cluster similarity relationships. Each term is represented by a circle, coloured by its cluster identity and scaled proportional to the number of genes involved.

Supplementary Table 1. Demographic characteristics of participants.

|  | **HCP-YA dataset** | | | |
| --- | --- | --- | --- | --- |
| **Variable** | **white Americans (N = 721)** | **African Americans (N = 101)** | ***T* value / Chi2 value** | ***P* value** |
| **Age (years)** | 28.97 (3.56) | 28.86 (3.59) | 0.29 | 0.77^a^ |
| **Sex (Male/Female)** | 343/378 | 44/57 | 0.42 | 0.52^b^ |
| **RMS** | 0.08 (0.02) | 0.09 (0.03) | -3.28 | 0.001^a^ |
| **ICV (****mm^3)** | 1.61e+06 (1.75e+05) | 1.51e+06 (1.60e+05) | 5.05 | <0.001^a^ |
| **Education level (years)** | 15.17 (1.65) | 14.36 (1.99) | 4.49 | <0.001^a^ |
| **Income** | 5.43 (2.01) | 3.98 (2.10) | 6.73 | <0.001^a^ |
| **Sleep score** | 4.52 (2.69) | 5.41 (2.69) | -3.11 | 0.002^a^ |
| **Substance use score** | 0.72 (0.66) | 0.58 (0.48) | 2.10 | 0.04^a^ |
| **Physical activity /Motor score** | 85.86 (5.29) | 83.50 (5.30) | 4.20 | <0.001^a^ |
| **Social relationship score** | 0.76 (6.24) | -0.92 (7.77) | 2.45 | 0.01^a^ |
|  | **HCP-D dataset** | | | |
|  | **white Americans (N = 404)** | **African Americans (N = 68)** | ***T* value / Chi2 value** | ***P* value** |
| **Age (months)** | 166.88 (45.85) | 202.46 (48.16) | -5.88 | <0.001^a^ |
| **Sex (Male/Female)** | 184/220 | 35/33 | 0.60 | 0.44^b^ |
| **RMS** | 0.08 (0.03) | 0.08 (0.03) | 1.04 | 0.30^a^ |
| **ICV (mm^3)** | 1.61e+06 (1.48e+05) | 1.58e+06 (1.95e+05) | 1.59 | 0.11^a^ |
| **Education level (years)** | 8.40 (4.35) | 11.71 (4.58) | -5.75 | <0.001^a^ |
| **Income** | 165279 (189848) | 65212 (56239) | 4.08 | <0.001^a^ |

Notes: Data are presented as means (standard deviations) for continuous variables and count for categorical variables. The cohort size was obtained after the image data quality control.
Composite scores for sleep, substance use, physical activity/motor, and social relationship were calculated as the average of the corresponding measures listed in Supplementary Table 2.
Household income was assessed using dataset-specific protocols. For the HCP-YA, total household income was coded on an ordinal scale: 1 = <$10,000; 2 = $10,000–$19,999; 3 = $20,000–$29,999; 4 = $30,000–$39,999; 5 = $40,000–$49,999; 6 = $50,000–$74,999; 7 = $75,000–$99,999; 8 = ≥$100,000. For the HCP-D, income represents the response to the question: "What was your best estimate of the total income of all family members from all sources, before taxes, in the last year?"
Abbreviations: RMS, root-mean-square framewise displacement; ICV, intracranial volume.
**^a^**. The p value is obtained by two-sample t-test (two-sided). **^b^**. The p value is obtained by Chi-square test.

Supplementary **Table 2. Lifestyle characteristics of participants.**

| **Behavioral domain** | **Formal name** | **Intuitive name** |
| --- | --- | --- |
| **Education** | SSAGA_Educ | Education |
| **Income** | SSAGA_Income | Income |
| **Sleep** | PSQI_Score | PSQI |
| **Substance use** | Num_Days_Drank_7days SSAGA_Alc_D4_Dp_Sx SSAGA_Alc_D4_Ab_Dx SSAGA_Alc_D4_Ab_Sx SSAGA_Alc_D4_Ab_Sx Num_Days_Used_Any_Tobacco_7days SSAGA_TB_Smoking_History SSAGA_TB_Still_Smoking SSAGA_Times_Used_Illicits SSAGA_Times_Used_Cocaine SSAGA_Times_Used_Hallucinogens SSAGA_Times_Used_Opiates SSAGA_Times_Used_Sedatives SSAGA_Times_Used_Stimulants SSAGA_Mj_Use SSAGA_Mj_Times_Used | Alcohol Use Alcohol Dependence (Sx) Alcohol Abuse (Dx) Alcohol Abuse (Sx) Alcohol Dependence (Dx) Tobacco Use Smoking History Current Smoking Illicits Use Cocaine Hallucinogens Opiates Sedatives Stimulants Marijuana History Marijuana |
| **Physical activity/Motor** | Endurance_Unadj GaitSpeed_Comp Dexterity_Unadj Strength_Unadj | Endurance GaitSpeed Dexterity Strength |
| **Social relationship** | Friendship_Unadj Loneliness_Unadj PercHostil_Unadj EmotSupp_Unadj InstruSupp_Unadj | Friendship Loneliness PercHostil EmotSupp InstruSupp |

**Supplementary Table 3**. **Representative enriched terms of the PLS1+ genes (Z > 5, DS > 0.1) in the HCP-YA dataset.** Log10 (*P_FDR_*) is the FDR adjusted *P*-value in log base 10. *P_spin_* denotes the *P*-value computed using a spin-based ensemble null model that accounts for the effects of gene coexpression and spatial autocorrelation.

| **Category** | **Enriched terms** | **Gene counts** | **Log10 (*P_FDR_*)** | ***P_Spin_*** |
| --- | --- | --- | --- | --- |
| Reactome Gene Sets | Nervous system development | 139 | -25.52 | <0.001 |
| GO Biological Processes | Regulation of trans-synaptic signaling | 126 | -21.09 | <0.001 |
| GO Biological Processes | Synapse organization | 99 | -20.06 | <0.001 |
| GO Biological Processes | Synaptic signaling | 117 | -19.81 | <0.001 |
| GO Biological Processes | Regulation of secretion by cell | 131 | -19.72 | <0.001 |
| GO Biological Processes | Neuron projection development | 148 | -18.17 | <0.001 |
| Reactome Gene Sets | Neuronal System | 98 | -16.75 | <0.001 |
| GO Biological Processes | Response to toxic substance | 69 | -14.23 | <0.001 |
| GO Biological Processes | Regulation of synapse structure or activity | 80 | -13.63 | <0.001 |
| Reactome Gene Sets | Adaptive Immune System | 139 | -13.44 | <0.001 |
| Reactome Gene Sets | Metabolism of amino acids and derivatives | 83 | -13.41 | <0.001 |
| GO Biological Processes | Behavior | 125 | -13.09 | <0.001 |
| GO Biological Processes | Import into cell | 130 | -12.63 | <0.001 |
| Reactome Gene Sets | Signaling by GPCR | 130 | -12.59 | <0.001 |
| GO Biological Processes | Small molecule biosynthetic process | 100 | -12.11 | <0.001 |
| GO Biological Processes | Brain development | 135 | -11.97 | <0.001 |
| KEGG Pathway | Neuroactive ligand signaling | 56 | -11.97 | <0.001 |
| Reactome Gene Sets | Cellular responses to stress | 138 | -11.81 | <0.001 |
| GO Biological Processes | Regulation of vesicle-mediated transport | 108 | -11.61 | <0.001 |
| GO Biological Processes | Localization within membrane | 108 | -11.08 | <0.001 |

**Supplementary Table 4**. Representative enriched terms of the PLS1+ genes (*Z* > 5, DS > 0) in the HCP-YA dataset. Log10 (*P_FDR_*) is the FDR adjusted *P*-value in log base 10. *P_spin_* denotes the *P*-value computed using a spin-based ensemble null model that accounts for the effects of gene coexpression and spatial autocorrelation.

| **Category** | **Enriched terms** | **Gene**  **counts** | **Log10 (*P_FDR_*)** | ***P_Spin_*** |
| --- | --- | --- | --- | --- |
| Reactome Gene Sets | Axon guidance | 143 | -27.3 | <0.001 |
| GO Biological Processes | Regulation of trans-synaptic signaling | 133 | -22.38 | <0.001 |
| GO Biological Processes | Synaptic signaling | 124 | -21.23 | <0.001 |
| GO Biological Processes | Neuron projection development | 161 | -21.23 | <0.001 |
| GO Biological Processes | Synapse organization | 103 | -20.58 | <0.001 |
| Reactome Gene Sets | Neuronal System | 108 | -20.04 | <0.001 |
| GO Biological Processes | Regulation of secretion by cell | 136 | -19.87 | <0.001 |
| GO Biological Processes | Import into cell | 141 | -14.72 | <0.001 |
| Reactome Gene Sets | Adaptive Immune System | 147 | -14.35 | <0.001 |
| GO Biological Processes | Regulation of synapse structure or activity | 84 | -14.34 | <0.001 |
| GO Biological Processes | Behavior | 132 | -13.87 | <0.001 |
| GO Biological Processes | Response to toxic substance | 70 | -13.71 | <0.001 |
| GO Biological Processes | Brain development | 145 | -13.53 | <0.001 |
| GO Biological Processes | Regulation of vesicle-mediated transport | 117 | -13.48 | <0.001 |
| Reactome Gene Sets | Metabolism of amino acids and derivatives | 85 | -13.08 | <0.001 |
| GO Biological Processes | Small molecule biosynthetic process | 106 | -13.07 | <0.001 |
| GO Biological Processes | Export from cell | 110 | -13 | <0.001 |
| Reactome Gene Sets | Cellular responses to stress | 147 | -12.98 | <0.001 |
| Reactome Gene Sets | Signaling by GPCR | 136 | -12.95 | <0.001 |
| GO Biological Processes | Localization within membrane | 116 | -12.53 | <0.001 |

**Supplementary Table 5**. **Representative enriched terms of the PLS1+ genes (Z > 5, DS > 0.2) in the HCP-YA dataset.** Log10 (*P_FDR_*) is the FDR adjusted *P*-value in log base 10. *P_spin_* denotes the *P*-value computed using a spin-based ensemble null model that accounts for the effects of gene coexpression and spatial autocorrelation.

| **Category** | **Enriched terms** | **Gene**  **counts** | **Log10 (*P_FDR_*)** | ***P_Spin_*** |
| --- | --- | --- | --- | --- |
| GO Biological Processes | Regulation of trans-synaptic signaling | 105 | -20.01 | <0.001 |
| GO Biological Processes | Synaptic signaling | 97 | -18.64 | <0.001 |
| Reactome Gene Sets | Axon guidance | 101 | -18.11 | <0.001 |
| GO Biological Processes | Neuron projection development | 122 | -17.74 | <0.001 |
| GO Biological Processes | Synapse organization | 78 | -16.4 | <0.001 |
| Reactome Gene Sets | Signaling by GPCR | 113 | -14.97 | <0.001 |
| Reactome Gene Sets | Neuronal System | 79 | -14.64 | <0.001 |
| GO Biological Processes | Regulation of secretion by cell | 99 | -14.54 | <0.001 |
| GO Biological Processes | Regulation of synapse structure or activity | 67 | -13.24 | <0.001 |
| GO Biological Processes | Regulation of membrane potential | 81 | -11.99 | <0.001 |
| GO Biological Processes | Behavior | 100 | -11.83 | <0.001 |
| GO Biological Processes | Response to toxic substance | 54 | -11.43 | <0.001 |
| GO Biological Processes | Brain development | 108 | -11.01 | <0.001 |
| GO Biological Processes | Actin filament-based process | 93 | -10.64 | <0.001 |
| GO Biological Processes | Regulation of system process | 89 | -10.08 | <0.001 |
| GO Biological Processes | Localization within membrane | 85 | -9.4 | <0.001 |
| GO Biological Processes | Cellular response to metal ion | 42 | -9.36 | <0.001 |
| GO Biological Processes | Regulation of monoatomic ion transport | 72 | -9.28 | <0.001 |
| GO Biological Processes | Export from cell | 79 | -9.1 | <0.001 |
| GO Biological Processes | Import into cell | 97 | -8.94 | <0.001 |

**Supplementary Table 6**. Representative enriched terms of the PLS1- genes (*Z* < -5, DS > 0.1) in the HCP-YA dataset. Log10 (*P_FDR_*) is the FDR adjusted *P*-value in log base 10. *P_spin_* denotes the *P*-value computed using a spin-based ensemble null model that accounts for the effects of gene coexpression and spatial autocorrelation.

| **Category** | **Enriched terms** | **Gene counts** | **Log10 (*P_FDR_*)** | ***P_Spin_*** |
| --- | --- | --- | --- | --- |
| GO Biological Processes | Metal ion transport | 125 | -11.99 | <0.001 |
| GO Biological Processes | Chromatin remodeling | 114 | -7.09 | <0.001 |
| GO Biological Processes | mRNA metabolic process | 109 | -6.9 | <0.001 |
| GO Biological Processes | Mitotic cell cycle | 99 | -6.31 | <0.001 |
| GO Biological Processes | Export from cell | 89 | -6.15 | <0.001 |
| GO Biological Processes | Potassium ion transmembrane transport | 42 | -6.04 | <0.001 |
| GO Biological Processes | Intracellular receptor signaling pathway | 55 | -5.75 | <0.001 |
| GO Biological Processes | Regulation of plasma membrane bounded cell projection organization | 103 | -5.71 | <0.001 |
| Reactome Gene Sets | Neuronal System | 72 | -5.37 | <0.001 |
| Reactome Gene Sets | Transport of small molecules | 109 | -5.36 | <0.001 |
| GO Biological Processes | DNA metabolic process | 112 | -4.86 | <0.001 |
| KEGG Pathway | Phosphatidylinositol signaling System | 27 | -4.48 | <0.001 |
| WikiPathways | Ciliopathies | 40 | -4.48 | <0.001 |
| GO Biological Processes | Regulation of cell morphogenesis | 49 | -4.47 | <0.001 |
| GO Biological Processes | Protein modification by small protein conjugation | 104 | -4.4 | <0.001 |
| GO Biological Processes | Protein complex oligomerization | 52 | -4.4 | <0.001 |
| GO Biological Processes | Regulation of small GTPase mediated signal transduction | 57 | -4.4 | <0.001 |
| GO Biological Processes | Lipid transport | 62 | -4.4 | <0.001 |
| KEGG Pathway | Calcium signaling pathway | 49 | -4.37 | <0.001 |
| KEGG Pathway | Adrenergic signaling in cardiomyocytes | 35 | -4.29 | <0.001 |

Supplementary **Table 7**. Representative enriched terms of the PLS1- genes (*Z* < -5, DS > 0) in the HCP-YA dataset. Log10 (*P_FDR_*) is the FDR adjusted *P*-value in log base 10. *P_spin_* denotes the *P*-value computed using a spin-based ensemble null model that accounts for the effects of gene coexpression and spatial autocorrelation.

| **Category** | **Enriched terms** | **Gene counts** | **Log10 (*P_FDR_*)** | ***P_Spin_*** |
| --- | --- | --- | --- | --- |
| GO Biological Processes | inorganic cation transmembrane transport | 142 | -12.87 | <0.001 |
| GO Biological Processes | chromatin remodeling | 128 | -8.35 | <0.001 |
| GO Biological Processes | mRNA metabolic process | 123 | -8.34 | <0.001 |
| Reactome Gene Sets | Transport of small molecules | 129 | -8.03 | <0.001 |
| GO Biological Processes | mitotic cell cycle | 109 | -6.78 | <0.001 |
| GO Biological Processes | export from cell | 98 | -6.66 | <0.001 |
| GO Biological Processes | potassium ion transmembrane transport | 46 | -6.66 | <0.001 |
| GO Biological Processes | protein modification by small protein conjugation | 123 | -6.66 | <0.001 |
| Reactome Gene Sets | DNA metabolic process | 127 | -5.96 | <0.001 |
| Reactome Gene Sets | lipid transport | 72 | -5.88 | <0.001 |
| GO Biological Processes | intracellular receptor signaling pathway | 59 | -5.85 | <0.001 |
| KEGG Pathway | import into cell | 119 | -5.78 | <0.001 |
| WikiPathways | regulation of cell projection organization | 114 | -5.69 | <0.001 |
| Reactome Gene Sets | Neuronal System | 77 | -5.18 | <0.001 |
| KEGG Pathway | Calcium signaling pathway | 55 | -5.12 | <0.001 |
| GO Biological Processes | cell morphogenesis | 119 | -5.05 | <0.001 |
| Reactome Gene Sets | Metabolism of lipids | 122 | -5.05 | <0.001 |
| GO Biological Processes | plasma membrane bounded cell projection assembly | 82 | -4.86 | <0.001 |
| GO Biological Processes | organophosphate biosynthetic process | 97 | -4.73 | <0.001 |
| GO Biological Processes | nucleobase-containing compound transport | 50 | -4.64 | <0.001 |

Supplementary **Table 8**. Representative enriched terms of the PLS1- genes (*Z* < -5, DS > 0.2) in the HCP-YA dataset. Log10 (*P_FDR_*) is the FDR adjusted *P*-value in log base 10. *P_spin_* denotes the *P*-value computed using a spin-based ensemble null model that accounts for the effects of gene coexpression and spatial autocorrelation.

| **Category** | **Enriched terms** | **Gene counts** | **Log10 (*P_FDR_*)** | ***P_Spin_*** |
| --- | --- | --- | --- | --- |
| GO Biological Processes | Metal ion transport | 97 | -13.21 | <0.001 |
| KEGG Pathway | Calcium signaling pathway | 42 | -6.19 | <0.001 |
| Reactome Gene Sets | Neuronal System | 55 | -5.57 | <0.001 |
| GO Biological Processes | Sodium ion transport | 36 | -5.57 | <0.001 |
| GO Biological Processes | Intracellular receptor signaling pathway | 41 | -5.19 | <0.001 |
| KEGG Pathway | cGMP-PKG signaling pathway | 30 | -4.76 | <0.001 |
| GO Biological Processes | Actin filament-based process | 70 | -4.66 | <0.001 |
| GO Biological Processes | Modulation of chemical synaptic transmission | 61 | -4.19 | <0.001 |
| GO Biological Processes | Cell morphogenesis | 77 | -4.19 | <0.001 |
| KEGG Pathway | Pathways in cancer | 61 | -4.14 | <0.001 |
| GO Biological Processes | Cell junction organization | 64 | -4.11 | <0.001 |
| GO Biological Processes | Regulation of plasma membrane bounded cell projection organization | 70 | -4.06 | <0.001 |
| GO Biological Processes | Brain development | 78 | -3.96 | <0.001 |
| WikiPathways | Dravet syndrome | 11 | -3.85 | <0.001 |
| GO Biological Processes | Embryonic morphogenesis | 66 | -3.84 | <0.001 |
| GO Biological Processes | Synaptic signaling | 56 | -3.78 | <0.001 |
| KEGG Pathway | MAPK signaling pathway | 40 | -3.78 | <0.001 |
| GO Biological Processes | Regulation of cell morphogenesis | 35 | -3.61 | <0.001 |
| GO Biological Processes | Protein phosphorylation | 43 | -3.61 | <0.001 |
| GO Biological Processes | Negative regulation of cellular component organization | 74 | -3.48 | <0.001 |

Supplementary **Table 9**. Representative enriched terms of the PLS1+ genes (*Z* > 5, DS > 0.1) in the HCP-D dataset. Log10 (*P_FDR_*) is the FDR adjusted *P*-value in log base 10. *P_spin_* denotes the *P*-value computed using a spin-based ensemble null model that accounts for the effects of gene coexpression and spatial autocorrelation.

| **Category** | **Enriched terms** | **Gene counts** | **Log10 (*P_FDR_*)** | ***P_Spin_*** |
| --- | --- | --- | --- | --- |
| GO Biological Processes | Synapse organization | 70 | -14.68 | <0.001 |
| GO Biological Processes | Modulation of chemical synaptic transmission | 86 | -14.68 | <0.001 |
| GO Biological Processes | Synaptic signaling | 80 | -13.76 | <0.001 |
| GO Biological Processes | Behavior | 95 | -13.27 | <0.001 |
| Reactome Gene Sets | Nervous system development | 84 | -12.56 | <0.001 |
| GO Biological Processes | Neuron projection development | 98 | -11.85 | <0.001 |
| Reactome Gene Sets | Neuronal System | 63 | -9.59 | <0.001 |
| Reactome Gene Sets | Signaling by GPCR | 90 | -9.5 | <0.001 |
| GO Biological Processes | Regulation of neuron projection development | 65 | -8.74 | <0.001 |
| GO Biological Processes | Regulation of secretion by cell | 77 | -8.55 | <0.001 |
| GO Biological Processes | Regulation of system process | 76 | -7.99 | <0.001 |
| GO Biological Processes | Response to toxic substance | 44 | -7.97 | <0.001 |
| GO Biological Processes | Monoatomic cation homeostasis | 67 | -7.22 | <0.001 |
| GO Biological Processes | Regulation of synapse structure or activity | 50 | -7.18 | <0.001 |
| GO Biological Processes | Negative regulation of cell population proliferation | 89 | -6.88 | <0.001 |
| GO Biological Processes | Regulation of growth | 75 | -6.88 | <0.001 |
| GO Biological Processes | Import into cell | 82 | -6.83 | <0.001 |
| GO Biological Processes | Gliogenesis | 45 | -6.82 | <0.001 |
| WikiPathways | Spinal cord injury | 27 | -6.71 | <0.001 |
| Reactome Gene Sets | Metabolism of amino acids and derivatives | 51 | -6.71 | <0.001 |

Supplementary **Table 10**. Representative enriched terms of the PLS1- genes (*Z* < -5, DS > 0.1) in the HCP-D dataset. Log10 (*P_FDR_*) is the FDR adjusted *P*-value in log base 10. *P_spin_* denotes the *P*-value computed using a spin-based ensemble null model that accounts for the effects of gene coexpression and spatial autocorrelation.

| **Category** | **Enriched terms** | **Gene counts** | **Log10 (*P_FDR_*)** | ***P_Spin_*** |
| --- | --- | --- | --- | --- |
| GO Biological Processes | Metal ion transport | 92 | -12.43 | <0.001 |
| GO Biological Processes | Potassium ion transmembrane transport | 33 | -6.46 | <0.001 |
| Reactome Gene Sets | Neuronal System | 54 | -5.96 | <0.001 |
| KEGG Pathway | Calcium signaling pathway | 40 | -5.91 | <0.001 |
| GO Biological Processes | Import into cell | 77 | -5.58 | <0.001 |
| KEGG Pathway | Pathways in cancer | 63 | -5.53 | <0.001 |
| GO Biological Processes | Export from cell | 62 | -5.53 | <0.001 |
| GO Biological Processes | Sodium ion transport | 34 | -5.22 | <0.001 |
| WikiPathways | Neuroinflammation and glutamatergic signaling | 27 | -5.22 | <0.001 |
| GO Biological Processes | Protein phosphorylation | 46 | -5.19 | <0.001 |
| WikiPathways | Dravet syndrome | 12 | -4.93 | <0.001 |
| GO Biological Processes | Cellular response to hormone stimulus | 63 | -4.88 | <0.001 |
| KEGG Pathway | Phosphatidylinositol signaling system | 21 | -4.51 | <0.001 |
| KEGG Pathway | Adrenergic signaling in cardiomyocytes | 27 | -4.5 | <0.001 |
| Reactome Gene Sets | Transport of small molecules | 73 | -4.34 | <0.001 |
| GO Biological Processes | Response to ketone | 34 | -4.34 | <0.001 |
| KEGG Pathway | MAPK signaling pathway | 40 | -4.32 | <0.001 |
| GO Biological Processes | Regulation of small GTPase mediated signal transduction | 41 | -4.24 | <0.001 |
| GO Biological Processes | Actin filament-based process | 65 | -4.21 | <0.001 |
| GO Biological Processes | Enzyme-linked receptor protein signaling pathway | 69 | -4.17 | <0.001 |

Equation for structural equation models

**Education:**Y_brain function_ ~ age + sex + RMS + ICV + c*group + b*education;
education ~ a*group + age + sex + RMS + ICV
**Substance Use:**Y_brain function_ ~ age + sex + RMS + ICV + SES + c*group + b*substance use;
substance use ~ a*group + age + sex + SES + RMS + ICV
**Income:**Y_brain function_ ~ age + sex + RMS + ICV + c*group + b*income;
income ~ a*group + age + sex + RMS + ICV
**Physical Activity:**Y_brain function_ ~ age + sex + RMS + ICV + SES + c*group + b*physical activity;
physical activity ~ a*group + age + sex + SES + RMS + ICV
**Social Relationships:**Y_brain function_ ~ age + sex + RMS + ICV + SES + c*group + b*social relationships;
social relationships ~ a*group + age + sex + SES + RMS + ICV
**Sleep:**Y_brain function_ ~ age + sex + RMS + ICV + SES + c*group + b*sleep;
sleep ~ a*group + age + sex + SES + RMS + ICV

**Supplemental References**

1. Van Essen DC, Smith SM, Barch DM, Behrens TE, Yacoub E, Ugurbil K, et al. The WU-Minn human connectome project: an overview. Neuroimage. 2013;80:62-79.

2. Harms MP, Somerville LH, Ances BM, Andersson J, Barch DM, Bastiani M, et al. Extending the Human Connectome Project across ages: Imaging protocols for the Lifespan Development and Aging projects. Neuroimage. 2018;183:972-984.

3. Li J, Bzdok D, Chen J, Tam A, Ooi LQR, Holmes AJ, et al. Cross-ethnicity/race generalization failure of behavioral prediction from resting-state functional connectivity. Sci Adv. 2022;8(11):eabj1812.

4. Yeo BT, Krienen FM, Sepulcre J, Sabuncu MR, Lashkari D, Hollinshead M, et al. The organization of the human cerebral cortex estimated by intrinsic functional connectivity. J Neurophysiol. 2011;106:1125-1165.
